# Sodium-Glucose Co-Transporter 2 Inhibitors and Hyperkalemia-Related Discontinuation of Renin-Angiotensin-Aldosterone System Inhibitors During Mineralocorticoid Receptor Antagonist Therapy: A Real-World Cohort Study

**DOI:** 10.3390/pharmacy14040091

**Published:** 2026-06-26

**Authors:** Abdullah Hashim Almalki, Nourah Abdulaziz Alorainan, Muhjah Abdulhakim Bukhari, Fahad Ali Dokhaikh, Salma Mohamed Abbas Quqandi, Reyan Hatem Merdad, Laila Fahad Sadagah

**Affiliations:** 1 College of Medicine, King Saud Bin Abdulaziz University for Health Sciences, Jeddah 11481, Saudi Arabia; 2 King Abdullah International Medical Research Center, Jeddah 22384, Saudi Arabia; 3 Nephrology Section, Department of Medicine, King Abdulaziz Medical City, Ministry of National Guard Health Affairs, Jeddah 11426, Saudi Arabia; 4 Nephrology Section, Department of Medicine, King Fahad Hospital, Al-Baha 65732, Saudi Arabia; 5 Endocrinology Section, Department of Medicine, King Abdulaziz Medical City, Ministry of National Guard Health Affairs, Jeddah 11426, Saudi Arabia

**Keywords:** hyperkalemia, RAASi, SGLT2 inhibitors, mineralocorticoid receptor antagonists, chronic kidney disease, heart failure, potassium, effect modification

## Abstract

**Background:** Hyperkalemia (HK) is a common complication of renin-angiotensin-aldosterone system inhibitor (RAASi) therapy, and the risk is often increased by concomitant use of a mineralocorticoid receptor antagonist (MRA). The effect of SGLT2i co-prescription on this risk in routine clinical practice remains incompletely understood. **Methods:** This is a secondary analysis of a published retrospective cohort of 905 adult RAASi users attending outpatient clinics at King Abdulaziz Medical City, Jeddah, Saudi Arabia (IRB: NRJ22J/279/11), followed for a median of 28 months. Patients were classified as RAASi alone (n = 723) or RAASi plus MRA (n = 182). Beta-blockers and digoxin were excluded from the exposure definition. Effect modification by SGLT2i was assessed using logistic regression with a multiplicative interaction term. **Results:** MRA addition was associated with significantly higher rates of any HK (48.4% vs. 28.9%; RR 1.67, 95% CI 1.38–2.02, *p* < 0.001) and moderate-to-severe HK (13.7% vs. 6.9%; RR 1.99, 95% CI 1.26–3.12, *p* = 0.003). Overall, RAASi discontinuation rates were similar between groups. SGLT2i co-prescription significantly modified the association between MRA use and HK-driven RAASi discontinuation (interaction *p* = 0.004): among patients without SGLT2i, MRA addition was associated with a more than 5-fold increase in HK-driven discontinuation (21.1% vs. 4.1%; RR 5.11, *p* = 0.001), whereas no significant excess risk was observed among SGLT2i users (1.8% vs. 4.2%; RR 0.44, 95% CI 0.12–1.57, *p* = 0.190), although this subgroup estimate was imprecise. CKD (aOR 2.16, 95% CI 1.56–2.99) and age ≥ 75 years (aOR 1.64, 95% CI 1.04–2.58) were the strongest independent predictors of HK. **Conclusions:** MRA addition to RAASi substantially increases HK burden, and SGLT2i co-prescription appears to protect against HK-driven RAASi discontinuation in combined RAASi–MRA-treated patients. In patients with established indications for SGLT2i, co-prescription may confer the additional benefit of preserving RAASi continuity in the setting of MRA combination therapy.

## 1. Introduction

Renin-angiotensin-aldosterone system inhibitors (RAASi), including angiotensin-converting enzyme inhibitors (ACEi), angiotensin receptor blockers (ARB), and angiotensin receptor-neprilysin inhibitors (ARNI), are widely used in cardiovascular and renal practice. These agents have proven benefits in conditions such as heart failure with reduced ejection fraction, diabetic nephropathy, and chronic kidney disease (CKD) with albuminuria, and their use is strongly recommended by international guidelines [1,2,3].

Hyperkalemia (HK), commonly defined as a serum potassium level above 5.1 mmol/L, remains a major challenge during RAASi therapy. Reported rates range from 15% to 40%, particularly among high-risk patients, including those with CKD, heart failure, and diabetes [4,5,6]. Beyond being a laboratory abnormality, HK may lead to serious cardiac arrhythmias and often prompts clinicians to reduce or discontinue RAASi therapy. Such interruptions may deprive patients of the cardiovascular and renal benefits these medications are intended to provide [7,8,9].

Mineralocorticoid receptor antagonists (MRA), including spironolactone and eplerenone, are commonly used as add-on therapy in patients with heart failure with reduced ejection fraction and resistant hypertension. However, MRAs can increase serum potassium levels through the inhibition of aldosterone-mediated potassium excretion, and combining them with RAASi is known to further increase the risk of HK [10]. In our previously published cohort of 905 RAASi users from King Abdulaziz Medical City, HK occurred in nearly one-third of patients prescribed RAASi, and MRA use was identified as an important medication-related risk factor. Recurrent HK episodes were also observed to occur at progressively shorter intervals [11].

In contrast, sodium-glucose co-transporter 2 inhibitors (SGLT2i) have been associated with lower serum potassium in clinical trials and observational studies [12,13]. This is likely related to their ability to promote urinary potassium excretion through increased distal sodium delivery and osmotic diuresis. Whether SGLT2i co-prescription can meaningfully reduce HK risk—and specifically enable RAASi continuity—in patients receiving combined RAASi–MRA therapy has not been systematically evaluated in real-world practice.

This study aimed to compare HK incidence, severity, and RAASi discontinuation between patients receiving RAASi alone and those receiving combined RAASi–MRA therapy. We also examined whether concomitant SGLT2i use modifies the relationship between MRA therapy and HK-related RAASi discontinuation and explored independent predictors of HK and RAASi discontinuation using multivariate analysis.

## 2. Materials and Methods

This is a secondary analysis of a retrospective cohort study conducted at King Abdulaziz Medical City (KAMC), a tertiary academic medical center in Jeddah, Saudi Arabia, the primary results of which have been published previously [11]. The study was approved by the King Abdullah International Medical Research Center (KAIMRC) under protocol number NRJ22J/279/11. Informed consent was waived per institutional policy for retrospective chart reviews.

We included all adult patients (aged ≥18 years) attending nephrology, cardiology, endocrinology, or general internal medicine outpatient clinics at KAMC between 1 October 2023 and 30 September 2024, who had a documented prescription of at least one RAASi within the preceding five years with evidence of active use, and who were followed for a minimum of six months. Full inclusion and exclusion criteria, data collection methods, and the primary outcomes of the cohort have been described in the primary publication.

Patients were classified into two groups based on concomitant MRA use: (1) RAASi alone (no MRA; n = 723), and (2) RAASi plus MRA (spironolactone or eplerenone; n = 182). SGLT2i use (empagliflozin, dapagliflozin, or canagliflozin) was assessed separately as a potential effect modifier.

The primary outcome was any hyperkalemia, defined as serum potassium > 5.1 mmol/L during follow-up. Secondary outcomes included the following: (1) HK severity (mild > 5.1–5.5, moderate > 5.5–6.0, or severe > 6.0 mmol/L); (2) RAASi discontinuation attributed to HK; (3) RAASi discontinuation for any reason; and (4) a composite adverse clinical outcome comprising emergency department visits, hospitalization, cardiac arrhythmia, or initiation of dialysis. RAASi discontinuation was attributed to hyperkalemia when the treating physician explicitly documented hyperkalemia as the reason for stopping RAASi in the electronic medical record, or when permanent discontinuation followed a documented hyperkalemia episode without an alternative documented cause; this attribution applied the same chart-review criteria used in the primary cohort study [11].

Continuous variables are reported as median (IQR) and compared using the Mann–Whitney U test. Categorical variables are expressed as frequencies and proportions, compared using chi-square or Fisher’s exact test. Relative risks (RRs) with 95% confidence intervals were computed using the log-binomial method with generalized linear modeling. Effect modification by SGLT2i was assessed by stratified analysis and formally tested using binary logistic regression with a multiplicative interaction term (MRA × SGLT2i), with significance determined by both the Wald test and likelihood ratio test (LRT). Multivariate binary logistic regression identified independent predictors of any HK and RAASi discontinuation; bootstrapping (2000 iterations) was used to derive 95% confidence intervals. A two-sided *p* < 0.05 was considered statistically significant. All analyses were performed using IBM SPSS Statistics, version 26.0 (IBM Corp., Armonk, NY, USA), as described in the primary report [11]. Because the present work is a secondary analysis of an existing cohort, the SGLT2i interaction analysis was not pre-specified in the primary study protocol and is reported as an exploratory, hypothesis-generating analysis.

Sex and gender were recorded as biological sex at the time of the clinic visit. Sex disaggregated data are presented in Table 1. No formal sex-stratified analysis was pre-specified, given the observational secondary design; however, sex differences in outcome rates were explored in descriptive analyses.

## 3. Results

### 3.1. Baseline Characteristics

A total of 905 patients were included. Of these, 723 (79.9%) were prescribed RAASi without MRA, and 182 (20.1%) received concomitant MRA. Baseline characteristics are presented in Table 1. Patients in the RAASi + MRA group were more likely to be male (74.7% vs. 46.8%), had a substantially higher prevalence of congestive heart failure (81.3% vs. 19.6%) and coronary artery disease (34.6% vs. 27.4%), and were more frequently prescribed ARNI (64.3% vs. 4.6%), consistent with an indication-driven prescribing pattern. SGLT2i use was markedly higher in the RAASi + MRA group (89.6% vs. 32.9%; *p* < 0.001). Baseline serum potassium and eGFR were similar between groups.

### 3.2. Hyperkalemia Incidence, Severity, and Clinical Outcomes

**Figure 1:**
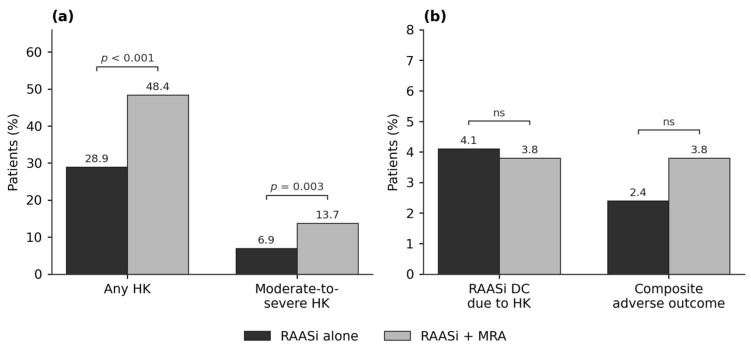
Hyperkalemia incidence, severity, and clinical outcomes by treatment group. (**a**) Proportion of patients with any hyperkalemia and with moderate-to-severe hyperkalemia in the RAASi-alone (n = 723) and RAASi + MRA (n = 182) groups. (**b**) Rates of RAASi discontinuation due to hyperkalemia and of the composite adverse outcome (emergency department visit, hospitalization, cardiac arrhythmia, or dialysis initiation). Exact between-group *p*-values are shown; ns, not significant. *DC*, discontinuation; *HK*, hyperkalemia; *MRA*, mineralocorticoid receptor antagonist; *RAASi*, renin-angiotensin-aldosterone system inhibitor.

MRA addition was associated with significantly higher rates of HK across all severity categories (Table 2, Figure 1). Any HK occurred in 48.4% of RAASi + MRA patients versus 28.9% in the RAASi-alone group (RR 1.67, 95% CI 1.38–2.02; *p* < 0.001). Moderate-to-severe HK was nearly twice as frequent in the MRA group (13.7% vs. 6.9%; RR 1.99, 95% CI 1.26–3.12; *p* = 0.003). RAASi discontinuation rates—both overall (8.2% vs. 8.7%) and due to HK specifically (3.8% vs. 4.1%)—were virtually identical between groups. The composite adverse clinical outcome was numerically higher in the RAASi + MRA group (3.8% vs. 2.4%) but did not reach statistical significance (RR 1.64, *p* = 0.262).

### 3.3. SGLT2i as an Effect Modifier of MRA-Associated HK Risk

**Figure 2:**
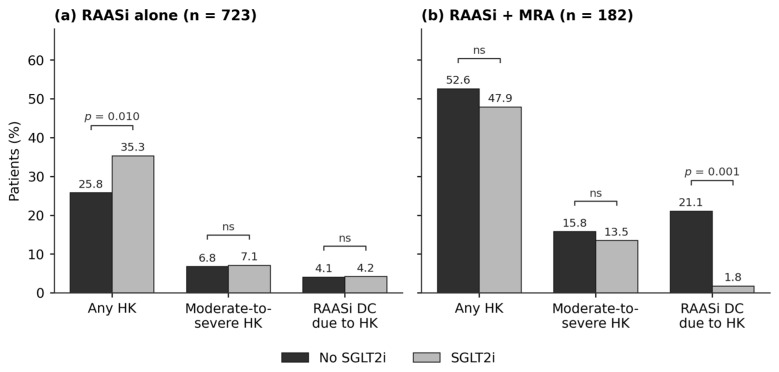
Effect of sodium-glucose co-transporter 2 inhibitor (SGLT2i) co-prescription on hyperkalemia outcomes, stratified by treatment group. (**a**) RAASi alone (n = 723) and (**b**) RAASi + MRA (n = 182): rates of any hyperkalemia, moderate-to-severe hyperkalemia, and RAASi discontinuation due to hyperkalemia among SGLT2i non-users versus users. In the RAASi + MRA group, discontinuation due to hyperkalemia was markedly lower among SGLT2i users (1.8% vs. 21.1%), indicating significant effect modification (interaction *p* = 0.004). Exact *p*-values are shown; ns, not significant (Fisher’s exact test). DC, discontinuation; HK, hyperkalemia; MRA, mineralocorticoid receptor antagonist; RAASi, renin-angiotensin-aldosterone system inhibitor.

Stratified analysis by SGLT2i co-prescription revealed a statistically significant interaction between MRA use and SGLT2i on HK-driven RAASi discontinuation (interaction OR 0.07, 95% CI 0.01–0.40; Wald *p* = 0.003; LRT *p* = 0.004). Results are summarized in Table 3 and illustrated in Figure 2.

In patients not receiving SGLT2i, MRA addition was associated with more than a 5-fold increase in RAASi discontinuation due to HK (4 of 19 [21.1%] vs. 20 of 485 [4.1%]; RR 5.11, 95% CI 1.93–13.48; *p* = 0.001). By contrast, among SGLT2i users, MRA addition was not associated with any excess discontinuation risk (3 of 163 [1.8%] vs. 10 of 238 [4.2%]; RR 0.44, 95% CI 0.12–1.57; *p* = 0.190). For any HK and moderate-to-severe HK, the interaction with SGLT2i was directionally consistent but did not reach statistical significance (interaction *p* = 0.212 and *p* = 0.753, respectively).

### 3.4. Multivariate Predictors of Hyperkalemia and RAASi Discontinuation

Multivariate logistic regression results are presented in Table 4. CKD (eGFR < 60 mL/min/1.73m^2^) was the strongest independent predictor of any HK (aOR 2.16; 95% CI 1.56–2.99; *p* < 0.001), followed by age ≥75 years (aOR 1.64; 95% CI 1.04–2.58; *p* = 0.032). MRA use showed a positive but non-significant association with HK after adjustment (aOR 1.49; *p* = 0.098), and SGLT2i use showed a trend toward lower discontinuation (aOR 0.46; 95% CI 0.20–1.06; *p* = 0.139), consistent with the interaction analysis.

## 4. Discussion

This secondary analysis of a real-world cohort of 905 RAASi users demonstrates that MRA add-on therapy is associated with a substantially higher rate of hyperkalemia, yet does not translate into increased overall RAASi discontinuation when SGLT2i is co-prescribed. The most clinically important finding is a statistically significant interaction between MRA use and SGLT2i co-prescription on HK-driven RAASi discontinuation (LRT *p* = 0.004): in the absence of SGLT2i, MRA addition conferred a more than 5-fold excess risk of HK-driven RAASi cessation, whereas no excess risk was evident among patients receiving concomitant SGLT2i, although the estimate in this stratum was based on few events (three discontinuations among 163 patients) and did not reach statistical significance. These findings extend the observations from our primary cohort report [11], which documented the overall HK burden and risk factors in this population, by identifying a clinically actionable pharmacological interaction.

The observation that MRA addition increases HK frequency is consistent with established pharmacology and prior evidence [10]. What is novel here is that, despite higher HK rates, MRA addition did not increase overall RAASi discontinuation and that this paradox is largely explained by the differential presence of SGLT2i. This finding is consistent with a large population-based Canadian cohort showing that SGLT2i initiation among RAASi users was associated with an 11% reduction in HK risk and significantly lower rates of RAASi discontinuation [12]. A network meta-analysis of randomized trial data similarly demonstrated that adding SGLT2i to the combination of MRA and RAASi markedly reduced HK occurrence compared with RAASi or MRA alone [13].

Several mechanisms may explain this observation. SGLT2i increase distal tubular sodium delivery, enhancing electrogenic potassium secretion in the cortical collecting duct, and promote osmotic diuresis that further dilutes luminal potassium concentration [14]. A meta-analysis of individual participant data from major randomized controlled trials confirmed that SGLT2i significantly reduce hyperkalemia risk in patients with type 2 diabetes on RAASi across a range of kidney function stages [15]. These effects are modest in absolute terms but may be sufficient to keep peak potassium below the threshold at which clinicians feel compelled to discontinue RAASi, which is consistent with our finding that the interaction is significant specifically for discontinuation rather than HK occurrence.

Avoiding unnecessary RAASi discontinuation is clinically important, particularly in CKD and heart failure populations. An observational study incorporating US and Japanese healthcare data demonstrated that HK-related RAASi down-titration or discontinuation was associated with significantly higher cardiorenal event rates compared with maintained therapy, even among patients who had experienced a hyperkalemia episode [16]. This evidence reinforces the premise that preserving RAASi continuity—particularly in patients with heart failure and CKD who are most dependent on these agents—is itself a clinically meaningful outcome.

CKD and older age emerged as the strongest independent predictors of HK in this cohort, consistent with the risk factors identified in our primary report [11] and the broader literature showing that reduced kidney function and older age confer the greatest HK susceptibility [17,18]. CKD impairs renal potassium excretion through reduced tubular secretory capacity, and, after multivariable adjustment, MRA use was no longer a statistically significant independent predictor of HK (aOR 1.49; 95% CI 0.93–2.39; *p* = 0.098). This attenuation is most consistent with collinearity between MRA use and its indications (heart failure and ARNI therapy) rather than an absence of pharmacological effect; nonetheless, the independent contribution of MRA cannot be precisely isolated in this observational design, and its role should be interpreted as supportive rather than definitive. For patients at the highest risk of HK-related RAASi loss, the combination of guideline-directed potassium binder use [19,20,21] and SGLT2i co-prescription may together constitute the most practical strategy to preserve RAASi therapy.

This study has several limitations that warrant a cautious interpretation. First, and most importantly, the key interaction finding rests on a small subgroup: only 19 patients received RAASi + MRA without SGLT2i, among whom 4 experienced HK-driven RAASi discontinuation. The resulting estimates are imprecise (interaction OR 0.07, 95% CI 0.01–0.40), and the protective association among SGLT2i users did not itself reach statistical significance (RR 0.44, *p* = 0.190); these results should therefore be regarded as hypothesis-generating rather than confirmatory. Second, the RAASi + MRA and RAASi-alone groups differed substantially at baseline—notably in heart failure (81.3% vs. 19.6%), ARNI use (64.3% vs. 4.6%), sex distribution, and SGLT2i use (89.6% vs. 32.9%)—reflecting indication-driven prescribing, so the comparison is in part between different patient populations. Although conventional multivariable adjustment was applied, the small number of discontinuation events precluded formal propensity-score modelling or matching, which would have been statistically unstable and could have conveyed false precision; reassurance is instead drawn from the consistency of the SGLT2i signal across the stratified, interaction-term, and multivariable analyses. Third, several determinants of potassium balance were unavailable, including dietary potassium intake, use of potassium binders, loop or thiazide diuretics, and medication adherence. Were SGLT2i users also more likely to receive binders or kaliuretic diuretics, residual confounding could account for part of the apparent protective association, biasing it away from the null. Fourth, median follow-up was shorter in the RAASi + MRA group (24.5 vs. 28.8 months); because shorter follow-up affords less time to accrue events, this difference would, if anything, bias the observed MRA-associated hyperkalemia excess toward the null, and approximate incidence-rate comparisons per patient-time were directionally concordant with the proportion-based estimates. Fifth, the timing of SGLT2i initiation relative to MRA exposure and to hyperkalemia events was not captured; without an established temporal sequence, the protective association may be vulnerable to immortal-time and exposure-misclassification bias. Sixth, as a secondary analysis of a previously published cohort, the interaction analysis was exploratory and not pre-specified. Finally, the single-center design limits generalizability. These limitations are inherent to the retrospective design shared with the primary report and are detailed therein.

## 5. Conclusions

MRA addition to RAASi substantially increases the burden of hyperkalemia, yet does not necessarily translate into greater RAASi discontinuation when SGLT2i is co-prescribed. SGLT2i use significantly modifies the effect of MRA addition on HK-driven RAASi discontinuation, with this protective interaction being biologically plausible but, given the small number of events, requiring confirmation in larger prospective studies. CKD and older age remain the strongest independent predictors of HK. These results suggest that SGLT2i co-prescription may help preserve RAASi continuity in high-risk patients.

## Figures and Tables

**Table 1:** Baseline characteristics of the study population by treatment group.

Characteristic	RAASi Alone (n = 723)	RAASi + MRA (n = 182)	*p*-Value
***Demographics***			
Age, years	60.5 (52.9–69.0)	62.8 (55.7–71.2)	0.054
Female sex	385 (53.2)	46 (25.3)	<0.001
Body mass index, kg/m^2^	30.6 (27.0–34.3)	29.6 (25.1–33.8)	0.062
***Comorbidities***			
Diabetes mellitus	565 (78.1)	146 (80.2)	0.554
Congestive heart failure	142 (19.6)	148 (81.3)	<0.001
Chronic kidney disease (eGFR < 60)	183 (25.3)	40 (22.0)	0.339
Coronary artery disease	198 (27.4)	63 (34.6)	0.048
Atrial fibrillation	39 (5.4)	15 (8.2)	0.152
***Laboratory parameters***			
eGFR, mL/min/1.73 m^2^	80.5 (61.5–96.0)	79.3 (63.2–92.4)	0.472
Serum potassium, mmol/L	4.4 (4.1–4.7)	4.3 (4.0–4.7)	0.382
***Medications***			
SGLT2 inhibitor	238 (32.9)	163 (89.6)	<0.001
ACE inhibitor	292 (40.4)	38 (20.9)	<0.001
Angiotensin receptor blocker	398 (55.0)	27 (14.8)	<0.001
Sacubitril/valsartan	33 (4.6)	117 (64.3)	<0.001
***Follow-up***			
Duration, months	28.8 (21.9–33.6)	24.5 (15.8–32.1)	0.003

Data are median (interquartile range) for continuous variables and n (%) for categorical variables. *p*-values are from the Mann–Whitney *U* test (continuous) or the chi-square test (categorical). *ACE*, angiotensin-converting enzyme; *eGFR*, estimated glomerular filtration rate; *MRA*, mineralocorticoid receptor antagonist; *RAASi*, renin-angiotensin-aldosterone system inhibitor; *SGLT2*, sodium-glucose co-transporter 2.

**Table 2:** Hyperkalemia outcomes and clinical events by treatment group.

Outcome	RAASi Alone (n = 723)	RAASi + MRA (n = 182)	RR (95% CI)	*p*-Value
Any HK (K^+^ > 5.1 mmol/L)	209 (28.9)	88 (48.4)	1.67 (1.38–2.02)	<0.001
Moderate-to-severe HK	50 (6.9)	25 (13.7)	1.99 (1.26–3.12)	0.003
RAASi discontinuation due to HK	30 (4.1)	7 (3.8)	0.93 (0.41–2.08)	0.854
RAASi discontinuation, any reason	63 (8.7)	15 (8.2)	0.95 (0.55–1.62)	0.839
Composite adverse outcome ^a^	17 (2.4)	7 (3.8)	1.64 (0.69–3.89)	0.262

^a^ Composite adverse outcome: emergency department visit, hospitalization, cardiac arrhythmia, or dialysis initiation. Data are n (%). HK, hyperkalemia; MRA, mineralocorticoid receptor antagonist; RAASi, renin-angiotensin-aldosterone system inhibitor; RR, relative risk.

**Table 3:** Effect of sodium-glucose co-transporter 2 inhibitor co-prescription on hyperkalemia outcomes, stratified by mineralocorticoid receptor antagonist use.

Outcome	RAASi Alone			RAASi + MRA		
	No SGLT2i (n = 485)	SGLT2i (n = 238)	*p*	No SGLT2i (n = 19)	SGLT2i (n = 163)	*p*
Any HK	125 (25.8)	84 (35.3)	0.010	10 (52.6)	78 (47.9)	0.799
Moderate-to-severe HK	33 (6.8)	17 (7.1)	0.895	3 (15.8)	22 (13.5)	0.799
RAASi discontinuation due to HK	20 (4.1)	10 (4.2)	1.000	4 (21.1)	3 (1.8)	0.001 *

Data are n (%). *p*-values are from Fisher’s exact test; * *p* < 0.05. For hyperkalemia-driven RAASi discontinuation, the multiplicative MRA × SGLT2i interaction was significant (interaction OR 0.07, 95% CI 0.01–0.40; Wald *p* = 0.003; likelihood ratio *p* = 0.004). HK, hyperkalemia; MRA, mineralocorticoid receptor antagonist; RAASi, renin-angiotensin-aldosterone system inhibitor; SGLT2i, sodium-glucose co-transporter 2 inhibitor.

**Table 4:** Univariate and multivariate predictors of hyperkalemia and of RAASi discontinuation due to hyperkalemia.

Variable	Univariate (Any HK)		Multivariate (Any HK)		Multivariate aOR for RAASi Discontinuation Due to HK (95% CI)
	OR (95% CI)	*p*	aOR (95% CI)	*p*	
Age ≥ 75 years	1.89 (1.23–2.88)	0.004 *	1.64 (1.04–2.58)	0.032 *	1.18 (0.30–2.79)
Diabetes mellitus	1.54 (1.08–2.19)	0.017 *	1.39 (0.95–2.04)	0.089	1.96 (0.82–9.87)
Congestive heart failure	2.04 (1.48–2.80)	<0.001 *	1.27 (0.84–1.92)	0.255	3.11 (1.23–7.01) *
CKD (eGFR < 60)	2.24 (1.64–3.05)	<0.001 *	2.16 (1.56–2.99)	<0.001 *	2.57 (1.23–5.27) *
MRA use	2.32 (1.67–3.24)	<0.001 *	1.49 (0.93–2.39)	0.098	0.75 (0.17–2.47)
SGLT2i use	1.88 (1.42–2.49)	<0.001 *	1.32 (0.94–1.86)	0.114	0.48 (0.14–1.30)

Data are OR or aOR (95% CI). Multivariate models used logistic regression with bootstrap 95% CIs (2000 iterations). * *p* < 0.05. aOR, adjusted odds ratio; CKD, chronic kidney disease; CI, confidence interval; eGFR, estimated glomerular filtration rate; HK, hyperkalemia; MRA, mineralocorticoid receptor antagonist; OR, odds ratio; RAASi, renin-angiotensin-aldosterone system inhibitor; SGLT2i, sodium-glucose co-transporter 2 inhibitor.

## Data Availability

The data that support the findings of this study are available from the corresponding author upon reasonable request. Data are not publicly available due to privacy and ethical restrictions.
